# A Pragmatic Test for Detecting Association between a Dichotomous Trait and the Genotypes of Affected Families, Controls and Independent Cases

**DOI:** 10.3389/fgene.2017.00049

**Published:** 2017-05-09

**Authors:** Meng Wang, William C. L. Stewart

**Affiliations:** ^1^The Research Institute at Nationwide Children's HospitalColumbus, OH, USA; ^2^Departments of Statistics and Pediatrics, Ohio State UniversityColumbus, OH, USA

**Keywords:** candidate gene association, meta-analysis, transmission disequilibrium, POPFAM+, sequencing

## Abstract

The efficient analysis of hybrid designs [e.g., affected families, controls, and (optionally) independent cases] is attractive because it should have increased power to detect associations between genetic variants and disease. However, the computational complexity of such an analysis is not trivial, especially when the data contain pedigrees of arbitrary size and structure. To address this concern, we developed a pragmatic test of association that summarizes all of the available evidence in certain hybrid designs, irrespective of pedigree size or structure. Under the null hypothesis of no association, our proposed test statistic (POPFAM+) is the quadratic form of two correlated tests: a population-based test (e.g., wQLS), and a family-based test (e.g., PDT). We use the parametric bootstrap in conjunction with an estimate of the correlation to compute *p*-values, and we illustrate the potential for increased power when (1) the heritability of the trait is high; and, (2) the marker-specific association is driven by the over-representation of risk alleles in cases, and by the preferential transmission of risk alleles from heterozygous parents to their affected offspring. Based on simulation, we show that type I error is controlled, and that POPFAM+ is more powerful than wQLS or PDT alone. In a real data application, we used POPFAM+ to analyze 43 genes of a hybrid epilepsy study containing 85 affected families, 80 independent cases, 234 controls, and 118 reference samples from the International HapMap Project. The results of our analysis identified a promising epilepsy candidate gene for follow-up sequencing: malic enzyme 2 (*ME2*; min *p* < 0.0084).

## Introduction

To boost the power of genetic association studies, researchers are invariably compelled to increase sample size (Hirschhorn and Daly, 2005). As such, there are effectively two options: (1) collect more independent cases and controls, or (2) integrate existing data on affected families (Nagelkerke et al., 2004; Epstein et al., 2005). Proponents of the former approach realize that any substantial increase in the number of cases and controls is likely to increase the rate of cryptic relatedness and the extent of unreported population substructure. Although, modern methods like GMMAT (Chen et al., 2016) and ROADTRIPS (Thornton and McPeek, 2010) can account for some (if not all) of these potential confounders while incorporating covariates and unphenotyped samples, these methods typically do not model the transmission of risk alleles from heterozygous parents to their affected offspring. As such, some statistical information is invariably lost. Proponents of the latter approach recognize that affected families are (1) robust to population substructure (reported or not) (Martin et al., 2000; Laird and Lange, 2006) which safeguards against spurious associations; and (2) genetically more homogeneous (on average) than cases with unknown family histories. Therefore, to realize the full potential of hybrid designs involving dichotomous traits with high heritability, affected families, controls, and (optionally) independent cases, researchers need an association test that is both statistically efficient and computationally tractable. In other words, “How can we extract the maximum amount of information in a reasonable amount of time from genotype data ascertained in different ways for a trait that is almost entirely genetic?”

Often, researchers employ the “divide and conquer” approach: that is, they analyze (possibly dependent) study-specific subsets of the data separately [e.g., a case-control analysis, and a family-based association analysis (Spielman et al., 1993; Laird et al., 2000)], where both designs may share an overlapping set of cases. While this approach is convenient, it can also be unsatisfying. For example, when one study-specific result is statistically significant but the other is not, the “divide and conquer” approach makes no attempt to summarize the overall evidence for association, and the correlation (if any) between study-specific results is typically ignored. Furthermore, this approach is statistically inefficient because the allele frequency information in related cases is usually ignored as well (Nagelkerke et al., 2004; Epstein et al., 2005; Greenberg et al., 2005).

Likelihood-based tests (Bourgain et al., 2003; Epstein et al., 2005; Thornton and McPeek, 2007) are popular alternatives to the “divide and conquer” approach, in part, because they produce a single test statistic that summarizes the overall evidence for association. However, these tests are statistically inefficient in that either the preferential transmission of risk alleles from heterozygous parents to their affected offspring is ignored (e.g., wQLS^1^, MQLS, etc.), or the limitations on data availability, ascertainment, family size, and/or structure (e.g., GDT^2^, SCOUT, CAPL etc.) can be severe.

A third approach uses “meta-analysis” to combine the separate study-specific results, and usually provides a more efficient summary of the evidence for association (Kazeem and Farrall, 2005; Chen and Lin, 2008). However, there are drawbacks to this approach as well. First, when the hybrid design involves affected families, a statistically efficient analysis is usually limited to small, nuclear families (e.g., case-parent triads). This means that large extended families must be decomposed into nuclear families or trios, which is inconvenient, inefficient, and may introduce bias. Second, correctly accounting for the correlation between individual study-specific results is generally not trivial. Third, although the weighted combination of study-specific results provides an effective solution (Putter et al., 2005; Bagos and Nikolopoulos, 2007; Chen and Lin, 2008; Mirea et al., 2012; Stewart and Cerise, 2013), the optimal weights are unknown as they depend on the marginal, but unknown, power of each component study (Won et al., 2009).

Here, we propose POPFAM+^3^, a pragmatic test that uses a quadratic form of population-based and family-based association tests to detect coherent alternatives (i.e., alternatives where risk alleles are over-represented in cases, and preferentially transmitted from heterozygous parents to their affected offspring). Because our test uses inheritance information on multiple levels (e.g., transmissions from heterozygous parents, identity-by-descent calculations among relatives, and the evolution of allele frequencies in the population), it outperforms several tests that use the “divide and conquer” approach and several likelihood-based tests as well. Furthermore, POPFAM+ can handle hybrid designs with extended families, and it is capable of detecting associations to common as well as low-frequency variants (e.g., SNPs with MAFs ≈ 0.025). POPFAM+ is at least as powerful as several commonly used meta-analysis methods, and the software is freely available online at: http://u.osu.edu/stewart.1212.

## Methods

For a given single nucleotide polymorphism (SNP) and for a dichotomous trait, let's suppose that we want to test the null hypothesis of no association. If the SNP is in LD (i.e., linkage disequilibrium) with a causal genetic factor, then the alleles at the SNP will be associated with the trait, and the allele frequency difference between cases and controls cannot be zero. It is also true that heterozygous parents will preferentially transmit risk alleles at the SNP to their affected offspring, thereby providing a second line of evidence for association. POPFAM+ combines these two lines of evidence to form a single, unified, and more powerful test of association.

Let (***G***,***P***) denote the genotypes and pedigree structures (i.e., biological relationships) of all individuals in a hybrid design. Recall that, for the purpose of this discussion, we are primarily concerned with hybrid designs containing affected families, controls, and (optionally) independent cases. Therefore, the genotypes in ***G*** can be decomposed into the genotypes of controls (***C***), independent cases (***K***), and affected families (***A***). For ease of exposition, we use a modified version^4^ of wQLS (denoted *X* ≡ *X*[***C***, ***K***, ***A***; ***P***]; Bourgain et al., 2003; Thornton and McPeek, 2007) to assess the difference in allele frequencies, and PDT^5^ (denoted *Y* ≡ *Y*[***A***; ***P***]; Martin et al., 2000, 2001) to assess the preferential transmission of risk alleles from heterozygous parents to their affected offspring. Essentially, wQLS is a quasi-likelihood score test that uses the kinship coefficients of relatives, which we computed with MERLIN^6^ (Abecasis et al., 2002), to account for genetic correlations within families. While PDT is a Wald-type test constructed from an M-estimator (Vaart, 1998), where the M-estimator is the sum of the genetic differences among (1) discordant sibling pairs, and (2) transmitted and untransmitted risk alleles. In practice however, any pair of population-based and family-based component test statistics can be used, provided that both tests are asymptotically *N*(0, 1) under the null hypothesis of no association. Here, we illustrate our proposed test with wQLS and PDT because these two component tests are applicable to a wide variety of hybrid designs involving dichotomous traits with high heritability. Under the null hypothesis, the limiting distribution of *X* and *Y* is bivariate normal (*BVN*) with mean *(EX, EY)*′ = **0** and variance-covariance matrix Σ. Note that both components: X (which is population-based) and Y (which is family-based) are correlated because both depend on ***A***—the genotypes in affected families. Furthermore, this correlation (denoted ρ) is estimated under the null hypothesis of no association by conventional gene dropping at the test SNP, where the only requirement for the simulation is a consistent estimate of the corresponding MAF (minor allele frequency).

In order to summarize the overall evidence for association, we combine *X* and *Y* through the following quadratic form:

(1) $$\begin{array}{c} \text{POPFAM}+\equiv (X,Y)\Sigma_{\tau }^{-1}(X,Y{)}^{\prime } \end{array}$$

where $\Sigma_{\tau }^{-1}$ is the inverse of $\Sigma_{\tau },\Sigma_{\tau }=\left(\begin{array}{cc} 1 & \rho \tau \\ \rho \tau & 1 \end{array}\right),$ and τ ∈ [0, 1] is a user-specified scaling factor for the off-diagonal terms of the variance-covariance matrix $\Sigma =\left(\begin{array}{cc} 1 & \rho \\ \rho & 1 \end{array}\right).$

Note that POPFAM+ does not follow a χ^2^ distribution with 2 degrees of freedom under the null hypothesis of no association. Therefore, we use the parametric bootstrap with $\hat{\rho }$ to estimate POPFAM+ *p*-values, and hence the type 1 error under the null. Specifically, we simulate (X^1^,Y^1^), …, (X^t^,Y^t^) from a $BVN\left(0,\hat{\Sigma }\right)$, and compute the limiting distribution of POPFAM+ empirically. Both PDT and wQLS can analyze data with missing genotypes and/or phenotypes, but neither method is particularly concerned with imputation, or integrating over missing data. Because POPFAM+ inherits these same properties, it too can be applied to hybrid designs with missing data.

**Figure 1:**
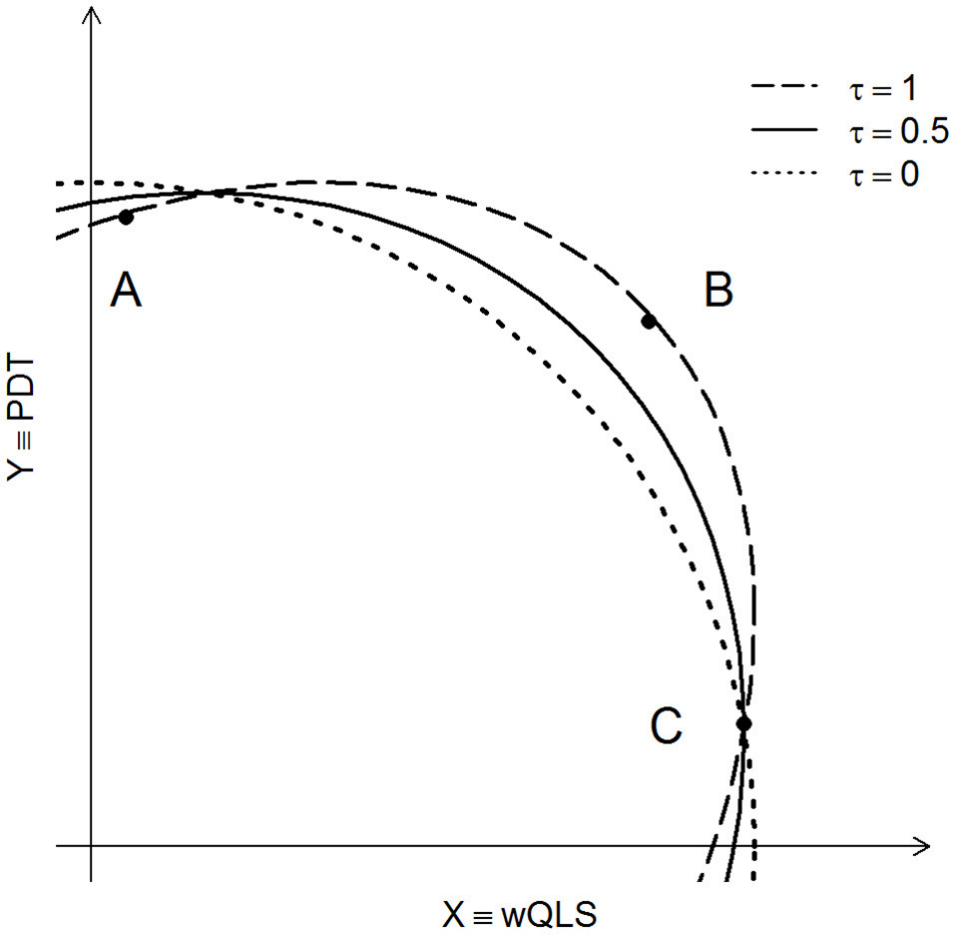
**Three contours of constant probability are shown for POPFAM+**. Dashed curve (τ = 1) is the density contour of the χ^2^ distribution with 2 degrees of freedom. The dotted line corresponds to τ = 0, and the solid line is the contour of constant probability for POPFAM+ when τ = 0.5. The X- and Y-axes are the values of (normalized) test statistics, with X representing the appropriately signed square root of wQLS, and Y representing PDT.

Ideally, we would like to use the best value of τ in Equation (1), but this would require knowledge of the joint distribution of (X, Y) under the alternative hypothesis of association. Because this information is never known, and because we found our results to be robust to different values of τ, we recommend setting τ = 0.5. In practice, this strikes a reasonable balance between a χ^2^ test with 2 degrees of freedom, and the test that defines “more extreme” in terms of Euclidean distance from the origin (see Figure 1 and Appendix A for more details).

To better understand the impact of τ, and how our proposed test compares with other competing tests (including a χ^2^ test with 2 degrees of freedom), let's consider a few instructive examples. In each example, we assume that the null hypothesis of no association is true, and that the sample is sufficiently large so that the finite sampling distribution of POPFAM+ is well approximated by its limiting distribution. First, if τ were 1, then the limiting distribution of POPFAM+ would be a χ^2^ distribution with 2 degrees of freedom. The contour of constant probability for this distribution is shown in Figure 1 (dashed line). Because points A, B, and C fall on this contour line, they have the same *p*-value (Figure 1). If instead, τ were 0, then POPFAM+ would use a notion of “more extreme” that corresponds to the idea of “more distant” from the origin, and hence the *p*-values at points A, B, and C would no longer be equal. In particular, the *p*-value at C would not change, but the *p*-value at A would increase and the *p*-value at B would decrease. Moreover, as the value of τ varies between 0 and 1, the contours of constant probability vary between dotted and dashed lines of Figure 1. Therefore, POPFAM+ has considerable flexibility, based on one's choice of τ, to detect coherent alternatives (i.e., alternatives where wQLS and PDT have the same sign, and neither is close to 0).

## Data description

We carried out extensive simulations to assess the performance of POPFAM+. Our simulations mimic the structure of an ongoing family study of idiopathic generalized epilepsy (IGE); summary statistics for the IGE families from this study are shown in Table 1. For each replicate we used the program SIMLA^7^ (Schmidt et al., 2005) to simulate genotype data on 47 case-parent triads, and 292 nuclear families where each nuclear family had at least two offspring. We considered a wide range of trait models (e.g., dominant and recessive, with relative risks of 2, 3, 5, and 10 for MAFs between 0.04 and 0.10). We also generated SNP genotypes for 100 independent controls. Among the case-parent triads, only those families with a positive history of disease (i.e., triads with at least two affected individuals) were included in the subsequent association analysis. And for the larger affected families, only those showing positive evidence for co-segregation (i.e., the maximum Kong and Cox lod > 0) were retained.

**Table 1:** **Summary statistics for families in simulated datasets**.

**Family size**	**Families w. a single affected offspring**	**Families w. multiple affected offspring**	**Total number of families**
3	47	0	47
4	117	9	126
5	74	15	89
6	39	9	48
7	27	2	29

*All affected families were ascertained on the basis of a single IGE (idiopathic generalized epilepsy) proband*.

When generating data under the null hypothesis of no association, D′—a measure of linkage disequilibrium between the test SNP and the disease gene, was fixed at zero. For all alternative hypotheses (i.e., when there was association), D′ was fixed at 0.8. For all scenarios, the MAFs at the disease gene and test SNP were the same, and these frequencies were varied from 0.04 to 0.10. Prevalence of the disease was fixed at 5%, while penetrance, relative risk, and MAFs were modified accordingly to satisfy this prevalence constraint. The results for each scenario under both null and alternative hypotheses were evaluated on the basis of 1,000 replicates with τ set to 0.5.

In a real data application, we analyzed 350 SNPs located in a 6 Mb region on chromosome 18 that was previously identified by cosegregation analysis (HLOD = 4.5; Durner et al., 2001) as a likely IGE locus. Our real data example, which is an extension of the Caucasian families in Greenberg et al. (2005), contains genotypes and phenotypes of 409 individuals from 83 affected families spanning multiple ethnicities. To reduce heterogeneity, we restricted attention to 32 families that showed positive evidence for cosegregation (maximum Kong and Cox LOD > 0). This data set also includes 27 independent Caucasian cases and 234 Caucasian controls. Furthermore, 118 ethnically matched (i.e., CEU) reference samples from the International HapMap Project (International HapMap Consortium, 2003) were included in the analysis. The affected families were analyzed with PDT, while all cases (both related and unrelated) and controls were analyzed with wQLS. For each SNP, we (1) estimated the MAF from the genotypes of controls; (2) simulated 1,000 replicates of the real data hybrid design (via gene dropping) under the null hypothesis of no association to estimate the correlation between PDT and wQLS (denoted ρ); (3) combined the observed PDT and the observed wQLS test statistics using the quadratic form in Equation (1) with τ set to 0.5; and (4) use the parametric bootstrap to estimate the corresponding POPFAM+ *p*-value based on 10,000 $BVN\left(0,\hat{\Sigma }\right)$ realizations.

## Results

To confirm the validity of POPFAM+, Table 2 shows the type I error for the MAFs considered (0.04, 0.07, and 0.10); the type I error for PDT and wQLS is shown as well. From Table 2, we see that the type I error of all three tests is ~5%.

**Table 2:** **Type I error for POPFAM+, PDT, and wQLS**.

	**MAF**		
	**0.04**	**0.07**	**0.1**
POPFAM+	0.051	0.052	0.046
wQLS	0.056	0.056	0.045
PDT	0.047	0.054	0.045

*Type I error for various simulation settings for POPFAM+, PDT, and wQLS. The 95% CIs for type I error for all scenarios considered included the nominal value 0.05*.

**Figure 2:**
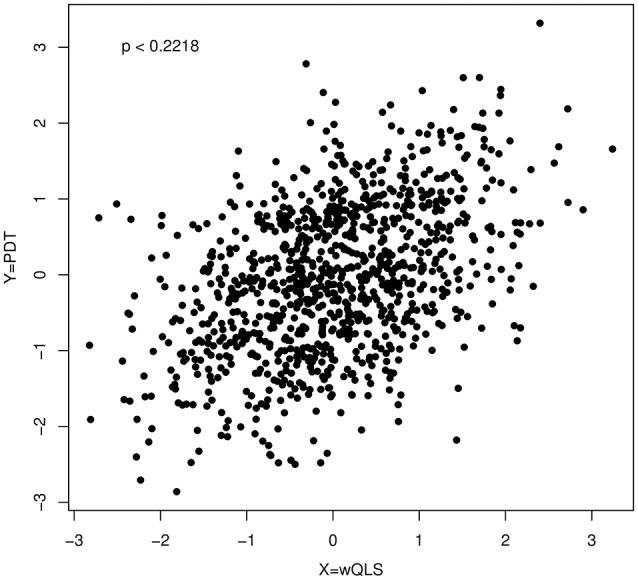
**A scatter plot of PDT and wQLS; the ***p***-value (***p*** = 0.2218) was obtained by applying a Kolmogorov–Smirnov test to the quadratic form in Equation (1) with τ equal to 1**.

Furthermore, Figure 2 shows a scatter plot of PDT and wQLS based on data simulated at a single SNP (D′ = 0, MAF = 10%, dominant model, 150 cases from 106 affected families containing 500 individuals, and 100 controls). For these data, we cannot reject the null hypothesis of bivariate normality (*p* = 0.2218).

**Figure 3:**
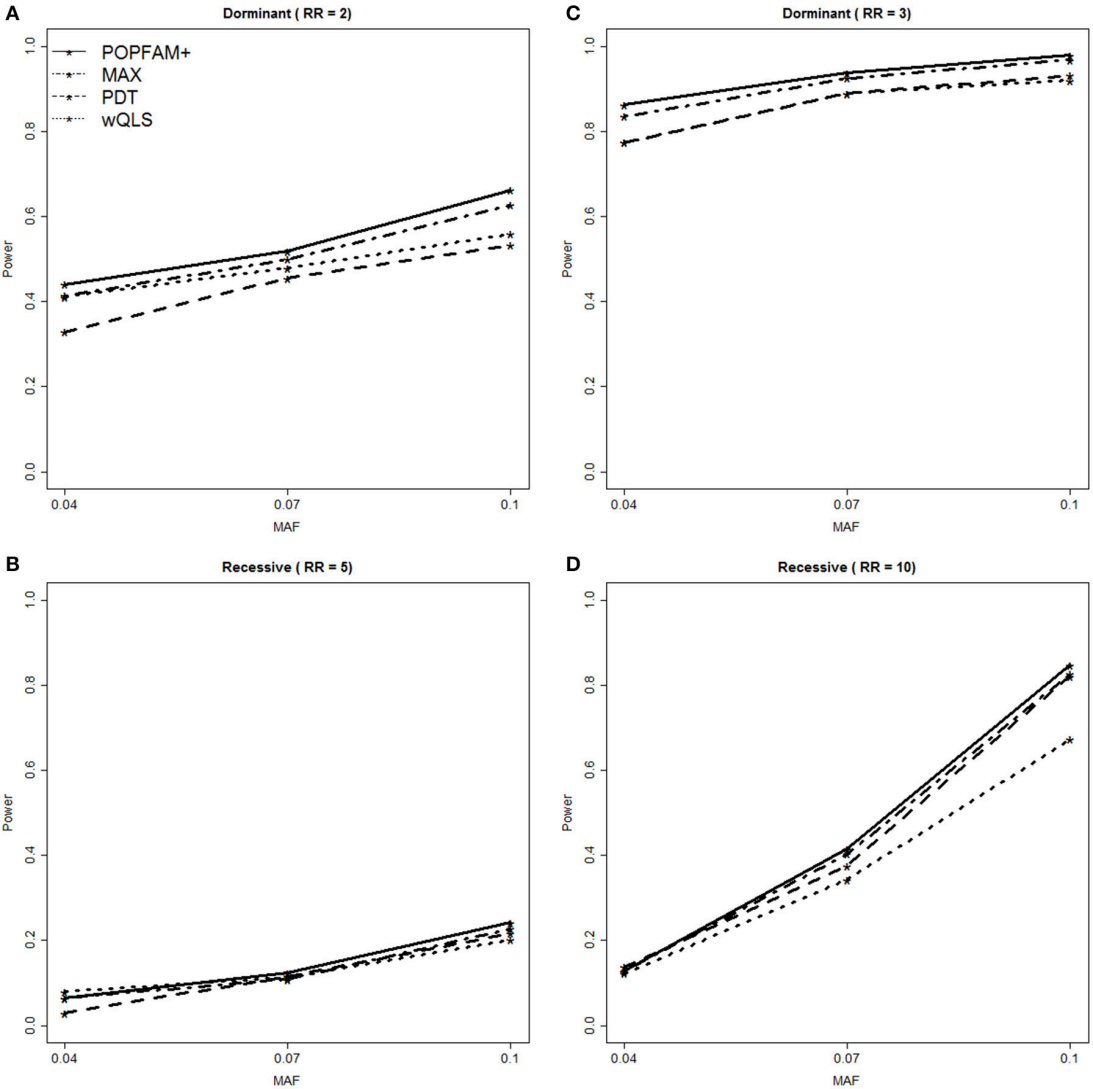
**Power Comparison for POPFAM+ (solid line), MAX (dash-dot), PDT (dash), and wQLS (dot)**. POPFAM+ and MAX have comparable power, and both outperform PDT and wQLS. Panel **(A)** shows power for a dominant model with relative risk (RR) of 2.0, while panel **(B)** shows power for RR = 3.0. Similarly, panel **(C)** shows power for a recessive model with RR = 5, while panel **(D)** shows power for RR = 10.

To compare the power of POPFAM+, PDT, wQLS, and the max(PDT, wQLS) (denoted MAX), we simulated and analyzed data for relative risks (RRs) of 2 and 3 for dominant models, and 5 and 10 for recessive models. The power results are summarized in Figure 3. For all of the alternative hypotheses considered, POPFAM+ had increased power compared to PDT and wQLS alone. For example, when the RR is 2 and the MAF is 0.1, POPFAM+ is 10% more powerful than either PDT or wQLS. In this same setting POPFAM+ has power comparable to MAX, with POPFAM+ at 66.2% [95% CI (63.3, 69.1%)] and MAX at 62.7% [95% CI (59.7, 65.7%)]. In all of the scenarios we considered, POPFAM+ is more powerful than the χ^2^ test with 2 degrees of freedom (data not shown); which is consistent with the finding of Joo et al. (2010) that MAX is more powerful than the χ^2^ test as well. Although, all five tests are statistically consistent when association is present (i.e., the power approaches 1.0 as the sample size increases), the relative gains in power achieved by POPFAM+ are important because power is typically far from 1.0 in most genetic studies of common complex traits.

In a real data application, we used POPFAM+ to analyze the genotypes and phenotypes of a complex hybrid design for IGE [*aka* Genetic Generalized Epilepsy (GGE)]. For each SNP in the 6 Mb candidate region, *p*-values based on POPFAM+ were computed. The top 10 genes ranked by minimum *p*-value (denoted min *p*) are shown in Table 3. For each gene, the minimum *p*-value is the smallest *p*-value among the SNP-specific *p*-values across the gene. For comparison, we also show the rank of each gene based on the International League Against Epilepsy (ILAE) meta-analysis (denoted ILAE; Anney et al., 2014), wQLS, and PDT (Table 3).

**Table 3:** **Top 10 genes ranked by POPFAM+, the ILAE Consortium, wQLS, and PDT**.

**POPFAM+**	**ILAE**	**wQLS**	**PDT**
CTIF	TXNL1	SETBP1	CTIF
SETBP1	PSTPIP2	ME2^*^	SERPINB5
ME2^*^	KATNAL2	SLC14A2	CFAP53
**SLC14A2**	**ZBTB7C**	RNF165	SMAD2
SERPINB5	**SLC14A2**	DYM	PSTPIP2
DYM	MEX3C	ST8SIA5	MYO5B
MYO5B	SKOR2	EPG5	SKOR2
**ZBTB7C**	LOXHD1	ZBTB7C	LOXHD1
ST8SIA5	SMAD7	MBD2	SETBP1
RNF165	HDHD2	CTIF	ZBTB7C

Genes with an asterisk (

* *) are epilepsy-related genes according to previous reports in the literature. Genes in bold are ranked in the top 10 by both POPFAM+ and ILAE*.

The results of our analysis identified a promising epilepsy candidate gene for follow-up sequencing: malic enzyme 2 (*ME2*; min_POPFAM+_ *p* < 0.0084, min_PDT_ *p* < 0.2851, min_wQLS_ *p* < 0.0024; Durner et al., 2001; Greenberg et al., 2005; Lee et al., 2007; Lucarelli et al., 2007). This illustrates the fact that POPFAM+ can detect coherent alternatives that a single component test might miss. For example, POPFAM+ detected *ME2*, despite the fact that PDT did not. We found four other potentially interesting genes that, to the best of our knowledge, are not epilepsy candidate genes: CBP80/20-Dependent Translation Initiation Factor (CTIF; min_POPFAM+_ *p* < 0.00238, min_PDT_ *p* < 0.0016, min_wQLS_ *p* < 0.0573), set binding protein (SETBP1; min_POPFAM+_ *p* < 0.0033, min_PDT_ *p* < 0.0587, min_wQLS_ *p* < 0.0023), Solute Carrier Family 14 (Urea Transporter), Member 2 (*SLC14A2*; min_POPFAM+_ *p* < 0.033, min_PDT_ *p* < 0.6376, min_wQLS_ *p* < 0.0092) and Zinc Finger and BTB Domain Containing 7C (*ZBTB7C*; min_POPFAM+_ *p* < 0.048, min_PDT_ *p* < 0.3173, min_wQLS_ *p* < 0.0428).

Because our real data analysis is fundamentally a fine-mapping study, we are not concerned with achieving statistical significance *per se*. Instead, we want to accurately prioritize of the genes beneath this 6 Mb cosegregation peak (Table 3). In other words, statistical significance for the 6 Mb region of interest was already achieved with our affected families (HLOD = 4.5), so what we want to know now is which gene within this region should we pursue first.

## Discussion

Compared to competing methods like PDT and wQLS, POPFAM+ has increased power to detect coherent alternatives (e.g., alternatives where risk alleles are over-represented in cases, and preferentially transmitted from heterozygous parents to their affected offspring). This could be particularly useful for detecting variants with MAFs < 5%, where the statistical power could be low unless sample sizes or effect sizes happen to be large (Lee et al., 2014). Note that, although MAX is not a linear combination of summary statistics, it is still a meta-analysis in that it can be computed solely from PDT and wQLS summary statistics. For all scenarios considered, POPFAM+ had comparable power to MAX (see Appendix A for more details), and to other linear combinations of PDT and wQLS, irrespective of whether MQLS—a competitor of wQLS, was used instead of wQLS (data not shown). From a meta-analysis perspective, the comparable performance of POPFAM+ is important because the best combination of summary statistics is not known (Won et al., 2009). Furthermore, although it is intuitively appealing to construct a linear combination from weights that vary in proportion to sample size, it is not clear how one would assess sample size in a meaningful way across the heterogeneous components of a hybrid design.

For several reasons, we fixed the scaling factor τ at 0.5 for all analyses. First, when little is known about the distribution of genetic effects, fixing τ at 0.5 strikes a nice balance between a χ^2^ test with 2 degrees of freedom, and the test that defines “more extreme” as more distant from the origin (Figure 1). Second, although our results were qualitatively similar with different values of τ (both simulated and real), POPFAM+ had increased power to detect associations (Figure 3) with τ = 0.5. Third, setting τ = 0.5 is equivalent to creating a composite rejection region, where a small fraction of the nominal type I error is attributed to a more stringent (but standard) rejection region, while the remaining fraction is attributed to a rejection region that favors coherent alternatives [i.e., sgn(PDT) = sgn(wQLS) and neither is close to zero; see Appendix B for details]. One could also interpret τ as a reflection of the strength of one's belief that coherent alternatives are more frequent than alternatives where PDT and wQLS have opposite signs. For example, if for some trait of interest a researcher believed that most alternatives fell in either quadrant I (PDT > 0, wQLS > 0) or quadrant III (PDT < 0, wQLS < 0), then a smaller value of τ may be more appropriate.

In principle, POPFAM+ could be extended to *n* > 2 lines of evidence, which for example, could facilitate the incorporation of gene expression data. Similarly, if researchers are concerned about the presence of population substructure then a program like STRUCTURE (Pritchard et al., 2000; Falush et al., 2003) could be used to first partition the hybrid data set into homogeneous subsets. Then, the respective POPFAM+ *p*-values from each subset could be combined using, for example, Fisher's method. Therefore, as we continue to move toward integrative genomics, POPFAM+ should give researchers an important, and computationally tractable, tool for detecting coherent alternatives between genotypes and common complex human phenotypes.

## Author contributions

MW: Methodology development, data analysis, manuscript drafting, and revisions. WS: Methodology conception and development, data acquisition, manuscript drafting, and revisions.

### Conflict of interest statement

The authors declare that the research was conducted in the absence of any commercial or financial relationships that could be construed as a potential conflict of interest.

## Figures and Tables

**Figure A1:**
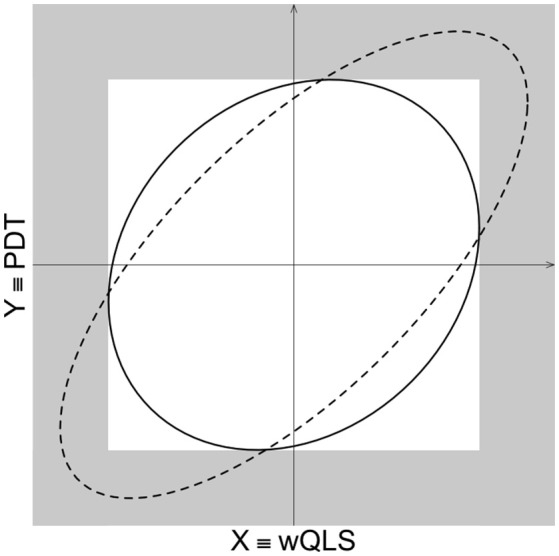
**Comparison of rejection regions for POPFAM+, MAX, and a χ^**2**^ with 2 degrees of freedom**. The rejection region of a POPFAM+ is outside the solid ellipse. The rejection region of χ^2^ test with 2 degrees of freedom is outside the dashed ellipse, and the rejection region of MAX, which is defined as max(X,Y), is shaded in gray.

**Figure A2:**
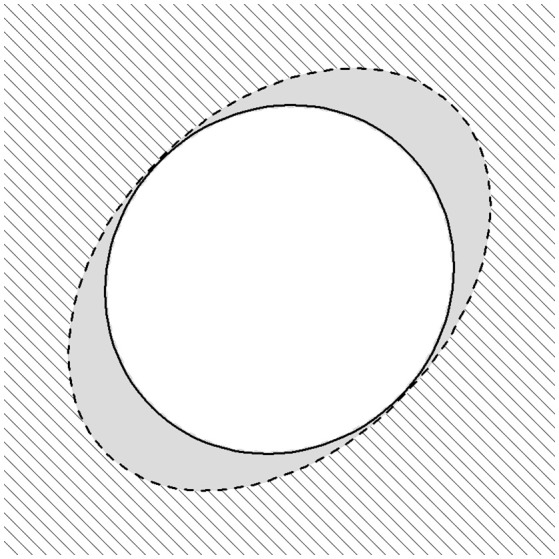
**The composite rejection region of POPFAM+**. The area outside the black dashed ellipse defines the rejection region of a χ^2^ test with 2 degrees of freedom with size 0.1α, while the area outside the solid ellipse defines a rejection region of size α for POPFAM+. Under the null hypothesis of “no association” the two twin crescents shaded in grey each have probability 0.45α, so that the total probability across all three regions: 0.1α, 0.45α, and 0.45α sum to α.

## Footnotes

^1^http://www.stat.uchicago.edu/~mcpeek/software/MQLS/download.html

^2^http://people.virginia.edu/~wc9c/GDT/Download.htm

^3^http://u.osu.edu/stewart.1212

^4^Because wQLS is a chi-squared statistic with 1 degree of freedom under the null hypothesis of no association, our modified version is sgn$\left({\hat{p}}_{case}-{\hat{p}}_{control}\right)*\sqrt{\text{wQLS}}$.

^5^http://hihg.med.miami.edu/software-download/pdt

^6^http://csg.sph.umich.edu/abecasis/Merlin/download/

^7^http://dmpi.duke.edu/simla-simulation-software-version-32.

## Appendix A: detecting coherent alternatives

For a fixed significance level (say 5%), the rejection regions for MAX (shaded gray), POPFAM+ (complement of the interior of the solid-line ellipse), and the χ^2^ test with 2 degrees of freedom (complements of the interior of the dash-line ellipse) are shown (Figure A1). Here (as in the text), *X* is wQLS and *Y* is PDT, so that the limiting distribution of *X* and *Y* under the null hypothesis of no association is *BVN* (bivariate normal) with mean *(EX, EY)*′ = **0** and variance-covariance matrix Σ. For any point on the perimeter of the white square, the *p*-value based on MAX is 5%, which means that MAX does not respect the *BVN* density of (X,Y). With respect to POPFAM+, the same is true for any point on the contour of the solid-line ellipse. Furthermore, just as POPFAM+ detects more coherent alternatives than the χ^2^ test with 2 degrees of freedom, so too does MAX.

It is also interesting to compare, the collection of points that give rise to the same *p*-value for POPFAM+ and MAX. Intuitively, such points help us to understand what the experimenter means by the phrase “more extreme.” In the case of MAX, the perimeter of the white square is a collection of points with the same *p*-value. This means that any point on the perimeter must (“in some sense”) be just as “extreme” as any other point on the perimeter. In contrast to Euclidean distance, where this statement is patently false (i.e., the corners are further from the origin than is the midpoint of any side), the statement is unequivocally true with respect to distance based on the infinity-norm. In this setting, the distance between the point (a,b) and the origin is max(|a|,|b|), and in this sense each point on the perimeter of the white square is in fact just as “extreme” as any other point on the perimeter. Now, let's consider the collection of points with equal *p*-values for POPFAM+ when τ equals zero. These points form a circle, which means that “more extreme” still corresponds to “further from the origin,” but now distance is measured with respect to the usual Euclidean metric. Therefore, τ = 0 can be viewed as a Euclidean analog to the MAX, which is why we consider values of τ in the range of [0, 1].

## Appendix B: composite rejection regions

To facilitate an understanding of composite rejection regions, consider Figure A2. Here, the rejection region for the χ^2^ test with 2 degrees of freedom of size 0.1 α is the complement of the interior of the dashed ellipse, and the rejection region for POPFAM+ with τ = 0.5 and size α is the complement of the interior of the solid ellipse. The rejection region of POPFAM+ can be partitioned into two regions: an area shaded with slanted lines which is the rejection region for a χ^2^ test with 2 degrees of freedom and size 0.1 α, and the two crescent shapes in gray. The area shaded with slanted lines contains 10% of the total type I error (e.g., 0.1 α), while the twin crescents contain the remaining fraction of the type I error (namely 0.9 α). Thus, the total type I error for POPFAM+ is still α.
